# Inflammatory and genomic interactions within keratoconus susceptible patients: a nationwide registered case–control study

**DOI:** 10.1186/s40662-024-00407-z

**Published:** 2024-10-02

**Authors:** Farideh Doroodgar, Fatemeh Alizadeh, Sana Niazi, Seyedeh Maryam Razavi, Nazanin Jalilian, Asaad Azarnezhad, Feizollah Niazi, Mohammad Ali Javadi, Jorge Alió del Barrio, Shima Dehghani, Majid Moshirfar, Zisis Gatzioufas, Renato Ambrósio, Jorge L. Alio

**Affiliations:** 1 Translational Ophthalmology Research Center, Tehran University of Medical Sciences, Tehran, Iran; 2 Negah Aref Ophthalmic Research Center, Shahid Beheshti University of Medical Sciences, Tehran, Iran; 3 Department of Genomic Psychiatry and Behavioural Genomics (DGPBG), School of Medicine, Roozbeh Hospital, Tehran University of Medical Sciences, Tehran, Iran; 4 Department of Clinical Biochemistry, School of Medicine, Kermanshah University of Medical Sciences, Kermanshah, Iran; 5 Liver and Digestive Research Center, Research Institute for Health Development, Kurdistan University of Medical Sciences, Sanandaj, Iran; 6 Faculty Member, Clinical Research Development Center, Shahid Modarres Educational Hospital, Shahid Beheshti University of Medical Sciences, Tehran, Iran; 7 Ophthalmic Research Center, Labbafinezhad Hospital, Shahid Beheshti University of Medical Sciences, Tehran, Iran; 8 Cornea, Cataract and Refractive Surgery Unit, Vissum Corporación, Alicante, Spain; 9 Bascom Palmer Eye Institute, University of Miami School of Medicine, Miami, FL USA; 10 John A. Moran Eye Center, University of Utah, Salt Lake City, UT USA; 11 Department of Ophthalmology, University Hospital Basel, Basel, Switzerland; 12 Department of Ophthalmology, Federal University of the State of Rio de Janeiro, Rio de Janeiro, Brazil; 13 Division of Ophthalmology, Universidad Miguel Hernández, Alicante, Spain; 14 Vissum Miranza Alicante, Calle Cabanal1, 03016 Alicante, Spain

**Keywords:** Cornea, Keratoconus, Interleukins, Genes

## Abstract

**Purpose:**

This study aimed to investigate the association between variants in the interleukin (IL)-1 gene cluster and susceptibility to keratoconus (KC) in an Iranian population.

**Methods:**

In the case group, there were 188 KC patients diagnosed by clinical findings and corneal tomography. The control group included all 205 healthy controls with no personal or family history of eye-related, metabolic, or immune system-related disease. Using the standard salting out extraction procedure, genomic DNA was isolated from peripheral blood leukocytes. The genotypes were determined by applying agarose gel electrophoresis for the IL-1RN 86 bp VNTR and polymerase chain reaction-restriction fragment length polymorphism (PCR–RFLP) for rs16944 and rs1143634.

**Results:**

The results showed a significant association between the IL-1β rs1143634 (rs1143634 T allele, *P* = 0.008) and IL-1RN 86 bp VNTR polymorphisms (LL and LS genotype, *P* = 0.048 and 0.012 respectively) and susceptibility to KC in the Iranian population. The genotype distributions of rs1143634 (*P* = 0.004) and rs2234663 (*P* = 0.042) significantly differed between case and control groups, with certain genotypes demonstrating a protective effect against KC. Logistic regression analysis revealed a protective effect of the IL-1RN L allele [odds ratio (OR) = 0.367, 95% confidence interval (CI): 0.240–0.562; *P* = 0.000] and certain haplotypes (OR = 0.628, 95% CI: 0.447–0.884; *P* = 0.007) against KC. However, no significant association was found for the IL-1β rs16944 polymorphism.

**Conclusion:**

This study provides evidence for an association between variants in the IL-1 gene cluster and susceptibility to KC in an Iranian population. Further research on larger and more diverse populations is warranted to validate these findings and explore the underlying mechanisms involved.

**Supplementary Information:**

The online version contains supplementary material available at 10.1186/s40662-024-00407-z.

## Background

Keratoconus (KC) is a multifactorial [1], bilateral and asymmetrical eye disease that leads to progressive thinning and steepening of the cornea [2]. This leads to irregular astigmatism and reduced visual acuity [3]. The prevalence of KC is estimated to be 50–2300 cases per 100,000 people affected by geologic variation, age groups, and screening technology [4–6] and 0.15% of the vision service plan enrollers in the United States [7]. A recent study from Yazd province, Iran, estimated the KC rate of incidence to be 22.3 to 24.9 per 100,000 people annually [8]. In the Middle East, the prevalence of KC in the Syrian student population was estimated at 1.43% [9].

The primary environmental and behavioral risk factors of KC pathogenesis are constant eye rubbing [10] and contact lens wear [11]. Several lines of evidence also point to a genetic determinant for KC [12]; for example, familial inheritance [13] and concordance between monozygotic twins as opposed to dizygotic twins [14]. KC is a convoluted attribute that presumably involves varying penetrance, several genes [15, 16] environmental influences, and different pathways involved [17]. Elevated proteinase activity [7, 11], reduced levels of proteinase inhibitors [13, 14, 17], exacerbated oxidative damage, and keratocyte apoptosis [18, 19] are among the different mechanisms involved in the pathogenesis of the disease.

Inflammation is an important factor in the pathogenesis of KC. However, the extent of its contribution to KC is not yet known [18]. A family of 11 cytokines called interleukin (IL)-1 controls immunologic and inflammatory responses, particularly during corneal wound healing. The two primary cytokines that are released after corneal injury are IL-1α and IL-1β, both of which cause remodeling by upregulating matrix metallopeptidase (MMPs) and inducing apoptosis. IL-1 is secreted by corneal epithelial cells and enters the stroma via the tear film [18]. IL-1RN is an antagonist of IL-1 that inhibits the activities of IL-1α and IL-1β and regulates several immunological and inflammatory reactions linked to IL-1, especially during the acute stage of infection and inflammation. IL-1α, IL-1β and IL-1RN are located as clusters on chromosome 2q14 [18, 19].

This study investigated the possible association between the IL-1 gene cluster and the presence of KC in the Iranian population. We performed a genetic association study for variants in the IL-1β (rs16944 and rs1143634) and intron 2 of IL-1RN (rs2234663, 86bp VNTR). We also evaluated these variants' linkage disequilibrium (LD) and allelic interactions. We successfully demonstrated a statistically significant association between IL-1β rs1143634 and IL-1RN 86bp VNTR and susceptibility to KC in the Iranian population.

## Methods

### Study design, clinical examination and sample collection

This case–control genetic association study was conducted in Negah Eye Hospital, Tehran, Iran, between June 2021 and April 2023. The Committees approved the study protocol for Ethics of Tehran University of Medical Sciences (IR.SBMU.ORC.REC.1399.015). Written informed consent was obtained from the study participants in line with the Declaration of Helsinki before their inclusion in the study.

In order to elucidate the relationship for both this study and subsequent immunogenetic studies, four groups of KC cases were formed, each representing a different degree of visual limitation, as follows: Stage 4: corrected distance visual acuity (CDVA) less than 20/400, logMAR units < 0.05; Stage 3: CDVA less than 20/100, logMAR units < 0.2; Stage 2: CDVA less than 20/40, logMAR units < 0.5; Stage 1: CDVA less than 20/20, logMAR units < 1; Stage 0: CDVA = 20/20, logMAR units = 1 [3, 20–22]. The Pentacam HR corneal tomography (Oculus, Wetzlar, Germany) and the Corvis ST (OCULUS Optikgeräte GmbH; Wetzlar, Germany) tests were done for all suspicious cases to detect mild forms of KC (Supplementary Tables 1 and 2) [2, 23–26]. Suspect cases were defined as stage 0. All cases were bilateral and diagnoses were approved by two cornea specialists (FD, MAJ). Complete medical histories were obtained from all patients with KC. No other ocular disorders or risk factors (including contact lens use, eye rubbing, atopy, etc.) or systematic diseases (including connective tissue disorders) were uncovered. All healthy controls had no personal or familial history of eye-related, metabolic, or immune system-related disease. All participants or their legal guardians signed consent forms.

### Isolation of genomic DNA and SNP genotyping

A K3EDTA tube was used to retain the 5 mL of peripheral blood drawn from the median cubital vein. Using the standard salting out extraction procedure, genomic DNA was isolated from peripheral blood leukocytes. The samples were then validated by electrophoresis on a 1% agarose gel, and their quantities were determined using ultraviolet (UV) spectroscopy and optical density ratios of 260/230 and 260/280. Polymerase chain reaction (PCR) and restriction fragment length polymorphism (RFLP) were used for genotyping of the targeted single nucleotide polymorphisms (SNPs). The initial stage of the PCR reactions was performed at 94 °C for 5 min before going through 35 cycles of 94 °C for 30 s, 58 °C for 30 s, 72 °C for 30 s, and an additional extension at 72 °C for 5 min. Ava1 and Tag1 restriction enzymes were used overnight to digest the target PCR product for rs16944 and rs1143634 polymorphisms, respectively. The processed PCR product underwent enzymatic digestion before being subjected to 2% agarose gel electrophoresis. By applying agarose gel electrophoresis to determine the copy number in the 86 bp-repeat, the IL-1RN 86 bp VNTR was evaluated. Supplementary Table 3 lists the forward and reverse primers, PCR products, and digested product lengths for the rs2234663, rs1143634, and rs16944 polymorphisms.

### Statistical analysis

The SPSS (version 20.0, SPSS Inc., Chicago, Illinois, USA) software was used to analyze the data. Using the Hardy–Weinberg genetic balancing test, the frequency of genotype distribution in the case and control groups was assessed. The *P* value and χ^2^ value were calculated using the Chi-squared test to examine the genotype and allele frequency distribution variations and KC. The odds ratio (OR) and its 95% confidence interval (CI) were calculated using binary logistic regression analysis to examine the relative risk of illness associated with genotypes or alleles at polymorphic locations. Haplotype analysis was also performed, and LD between the SNPs was evaluated using the SNPAnalyzer software. Protective/predisposing effects of haplotypes were estimated by logistic regression analysis. Differences with *P* values less than 0.05 were regarded as statistically significant for all two-sided probability tests.

## Results

Three hundred and ninety-three Iranian subjects were enrolled in this study, consisting of 205 healthy individuals (sex, 106 males and 99 females; age, mean ± SD: 33.26 ± 7.04 years) and 188 KC patients (sex, 97 males and 91 females; age, mean ± SD: 31.78 ± 9.18 years). The mean age distribution of the case and control groups did not differ substantially, and the demographics (age and sex) were evenly distributed across males and females (*P* = 0.982 and *P* = 0.077, respectively).

The results in Table 1 demonstrate the pertinent data of the genotype distribution of IL-1β gene rs1143634, rs16944, and IL-1RN VNTR polymorphisms. Although the genotype frequency distribution of rs2234663 for the case and control groups was not in the Hardy–Weinberg equilibrium (HWE) (*P* < 0.05, Table 1), rs1143634 and rs16944 genotype distribution were in the HWE (*P* > 0.05, Table 1). Subgroup analyses by age and sex were also performed. The genotype distributions of rs1143634 in men/women of more than 35 years of age were statistically different between case and control groups (*P* < 0.05 and *P* = 0.034, respectively). The genotype distributions of rs2234663 were significantly different only in the men's group (*P* = 0.016). Subgroup analysis based on sex and age for rs16944 revealed no statistically significant genotype distribution between case and control groups.

**Table 1:** Genotype distribution of IL-1RN 86 bp VNTR and IL-1β gene rs1143634, rs16944 polymorphisms in keratoconus cases and healthy controls

rs2234663 (86 bp VNTR)					rs16944 (T > C)					rs1143634 (C > T)				
		Control	Case	*P* value	*IL -1β*		Control	Case	*P* value	*IL -1β*		Control	Case	*P* value
Female	LL	75	66	0.463	Female	CC	29	22	0.084	Female	CC	55	37	**0.021**
	LS	18	10			TC	54	42			TC	35	50	
	SS	5	6			TT	16	27			TT	9	4	
Male	LL	80	58	**0.016**	Male	CC	25	23	0.581	Male	CC	65	42	**0.011**
	LS	18	21			TC	54	55			TC	40	49	
	SS	7	18			TT	27	19			TT	1	6	
< 35 years	LL	100	83	0.216	< 35 years	CC	35	29	0.718	< 35 years	CC	76	55	0.064
	LS	25	23			TC	71	70			TC	52	67	
	SS	9	16			TT	28	31			TT	6	8	
> 35 years	LL	55	41	0.159	> 35 years	CC	19	16	0.773	> 35 years	CC	44	24	**0.034**
	LS	11	8			TC	37	27			TC	23	32	
	SS	3	8			TT	15	15			TT	4	2	
Total	LL	155	124	**0.042**	Total	CC	54	45	0.679	Total	CC	120	79	**0.004**
	LS	36	31			TC	108	97			TC	75	99	
	SS	12	24			TT	43	46			TT	10	10	
HWE	*P* = 0.000				HWE		*P* = 0.416			HWE		*P* = 0.693		

*HWE* = Hardy–Weinberg equilibrium

*P* values in bold indicate statistical significance

### Association of rs2234663 (IL-1RN 86 bp VNTR) and IL-1β gene rs1143634 and rs16944 polymorphisms with KC

Tables 2, 3, and 4 show the logistic regression analysis and frequency distribution of the rs2234663 VNTR of IL-1RN, rs1143634, and rs16944 polymorphisms of the IL-1β gene in KC and the control group. Based on the differences of 86-bp VNTR, different alleles were expected. However, only IL-1RN-S (2-repeats) and IL-1RN-L (4 and 5 repeats) were detected in this study. We did not detect IL-1RN VNTR of 1, 3 and 6 replications in our population. The frequency distribution of LS heterozygous and LL homozygous genotypes significantly differed between the KC cases and the control group (*P* = 0.048 and *P* = 0.012, respectively). Indeed, the genotypes LS and LL were found to exert a protective effect against KC (OR = 0.431, 95% CI: 0.185–1.000; OR = 0.4, 95% CI: 0.192–0.832). The findings also revealed that, in comparison to the frequency distribution of the S allele in rs2234663, the frequency distribution of the L allele was lower in the case group than in the control group (77.9% vs. 90.6%), the difference of which was statistically significant (*P* = 0.000) and revealed a protective effect for the allele L of rs2234663 polymorphism against KC (OR = 0.367, 95% CI: 0.240–0.562).

**Table 2:** Association of IL -1RN 86 bp VNTR with keratoconus cases

Genotype	Cases (n = 188)	Controls (n = 205)	OR	95% CI	*P*
	n (%)	n (%)			
rs2234663					
SS	24 (13.4%)	12 (5.9%)	1.000	–	–
LS	31 (17.3%)	36 (17.7%)	0.431	0.185–1.000	**0.048**
LL	124 (69.3%)	155 (76.4%)	0.400	0.192–0.832	**0.012**
S	79 (22.1%)	36 (9.4%)	1.000	–	–
L	279 (77.9%)	346 (90.6%)	0.367	0.240–0.562	**0.000**
Recessive (LL vs. LS + SS)	123 (44.1%)	156 (55.9%)	0.688	0.437–1.083	0.105
Dominant (LL + LS vs. SS)	154 (44.5%)	192 (55.5%)	0.401	0.194–0.828	**0.011**
Codominant (LS vs. LL + SS)	147 (46.7%)	168 (53.3%)	0.984	0.580–1.670	0.953

*OR* = odds ratio; *CI* = confidence interval; *P* values in bold indicate statistical significance

**Table 3:** Association of IL-1β gene rs1143634 polymorphism with keratoconus cases

Genotype	Cases (n = 188)	Controls (n = 205)	OR	95% CI	*P*
	n (%)	n (%)			
rs1143634 (C > T)					
CC	79 (42.0%)	120 (58.5%)	1.000	–	–
TC	99 (52.7%)	75 (36.6%)	1.320	0.523–3.334	0.556
TT	10 (5.3%)	10 (4.9%)	1.510	0.605–3.817	0.605
C	257 (68.3%)	315 (76.8%)	1.000	–	–
T	119 (31.6%)	95 (23.2%)	1.530	1.119–2.106	**0.008**
Recessive (TT vs. CC + TC)	178 (47.7%)	195 (52.3%)	0.913	0.371–2.245	0.842
Dominant (TT + TC vs. CC)	109 (56.1%)	85 (48.9%)	1.948	1.304–2.910	**0.001**
Co-dominant (TC vs. CC + TT)	89 (40.6%)	130 (59.4%)	1.928	1.288–2.886	**0.001**

*OR* = odds ratio; *CI* = confidence interval

*P* values in bold indicate statistical significance

**Table 4:** Association of IL-1β gene rs16944 polymorphism with keratoconus cases

Genotype	Cases (n = 188)	Controls (n = 205)	OR	95% CI	*P*
	n (%)	n (%)			
rs16944 (T > C)					
CC	45 (23.9%)	54 (26.3%)	1.00	–	–
TC	97 (51.6%)	108 (52.7%)	1.07	0.66–1.74	0.76
TT	46 (24.5%)	43 (21.0%)	1.28	0.72–2.27	0.39
C	187 (49.7%)	216 (52.7%)	1.00	–	–
T	189 (50.3%)	194 (47.3%)	1.12	0.85–1.48	0.40
Recessive (TT vs*.* CC + TC)	142 (75.5%)	162 (79.0%)	1.22	0.76–1.95	0.40
Dominant (TT + TC vs. CC)	143 (75.5%)	151 (73.6%)	1.13	0.71–1.79	0.58
Co-dominant (CT vs. CC + TT)	91 (48.4%)	97 (47.3%)	0.96	0.644–1.423	0.83

*OR* = odds ratio; *CI* = confidence interval

In the dominant model, the genotype distribution of LS + LL in the case–control group was statistically significant when different inheritance modes were taken into account (*P* = 0.011), and the regression analysis showed a protective role of this inheritance model (OR = 0.401, 95% CI: 0.194–0.828). However, in the recessive and codominant models, the frequency distribution was not significantly different (*P* = 0.105, *P* = 0.953, respectively).

Chi-squared test and logistic regression were applied to investigate the genotype/allele distribution and association of IL-1β gene rs1143634 SNP with KC risk. As indicated in Table 3, the results disclosed that the IL-1β rs1143634 allele had a significantly different distribution in cases compared to controls. However, TC and TT genotype distribution were not significantly different between case and control groups, where the allele C and genotype CC were considered the reference wild type allele/genotype. Our findings showed that individuals with allele T have an increased risk for KC disorder (OR = 1.53, 95% CI: 1.119–2.106; *P* = 0.008). Furthermore, the model analysis revealed that the carriers of the dominance model (TC + TT vs. CC) and codominant model (TC vs. TT + CC) had an increased risk of KC (*P* = 0.001). However, we found that carriers of the recessive model (TT vs. TC + CC) had no real relationship with the other KC progression risk factors (*P* > 0.05).

The variation between the two groups, using CC as the reference type, was not statistically significant for both the TC and CC genotypes with regards to the frequency distribution and logistic regression analysis of the IL-1β gene rs16944 (Table 4, *P* > 0.05). Logistic regression analysis revealed no protective/predisposing association with KC in the studied population CC and TC genotypes. Additionally, the study of several inheritance models revealed no variance in the frequency distribution of the CC and TC genotypes that could be considered statistically significant, and thus, following logistic regression analysis, there was no significant correlation with the examined disease (*P* > 0.05). Allele distribution was also not statistically different in cases compared to controls, and accordingly, a significant association was not observed with KC in study participants.

### Haplotype analysis

The frequency of haplotypes (from left to right rs16944, rs1143634, and rs2234663) was assessed using SNPanalyzer (Table 5). We found eight haplotypes (h1–h8), where the h1 with CTL sequence was the most frequent haplotype (0.348). We further analyzed haplotypes to investigate whether any haplotype was linked to KC (Table 5). Because of the low frequency of h8 (0.009), it was omitted from downstream analysis. Considering haplotypes with frequencies of ≥ 1%, logistic regression analysis indicated a haplotype effect for h2 and h7. Our data revealed that haplotype h2 carriers had a lower risk of KC (OR = 0.628, 95% CI: 0.447–0.884; *P* = 0.007). In contrast, the h7 haplotype significantly increased the risk of KC (OR = 2.448, 95% CI: 1.35–4.437; *P* = 0.002]. We also performed LD analysis of the selected polymorphisms. Supplementary Fig. 1 shows the haplotype block formed by the LD study, which contains the two SNPs rs16944 and rs1143634 [D′ = 0.49216, r^2^ = 0.08612, logarithm of the odds (LOD) = 32.61988, *P* = 0.000).

**Table 5:** Distribution of different haplotypes of IL-1 cluster variants (rs16944, rs1143634, and rs2234663) and the association with keratoconus

Haplotype	Sequence	Frequency
h1	CTL	0.348
h2	CCL	0.250
h3	TCL	0.159
h4	CTS	0.072
h5	TTL	0.057
h6	CCS	0.056
h7	TCS	0.045
h8	TTS	0.009

Analysis model	Haplotype	χ^2^	*P* value	OR	Lower CI of OR	Higher CI of OR
Multiplicative	h1	0.245	0.620	0.93	0.698	1.239
	h2	7.177	**0.007**	0.628	0.447	0.884
	h3	0.059	0.809	1.048	0.717	1.533
	h4	1.935	0.164	1.551	0.832	2.891
	h5	1.353	0.245	1.535	0.742	3.178
	h6	0.372	0.542	0.83	0.455	1.513
	h7	9.19	**0.002**	2.448	1.35	4.437
	h8	1.521	0.218	0	0	0

*OR* = odds ratio; *CI* = confidence interval; *P* values in bold indicate statistical significance

## Discussion

Apoptosis is considered an important process in KC pathogenesis. Indeed, KC corneas exhibit a hyper apoptotic phenotype [27], the apoptosis in keratocytes is thought to be triggered by IL-1. IL-1 is released in response to chronic mechanical injury to the cornea (e.g., eye rubbing) [28]. Since a genetic predisposition to the disease allows an environmental trigger to cause an inflammatory component, we examined whether variants in the IL-1 gene cluster confer susceptibility to KC in the Iranian population.

This study found a strong protective role for the IL-1RN L allele against KC. The genotypes LL and LS were associated with a reduced risk of KC (OR = 0.4 and 0.431, respectively). This association was also seen with a dominant mode of inheritance. However, no significant association between IL-1RN VNTR and the likelihood of KC was found in other experiments by Kim et al. and Palamar et al. [29, 30]. The difference, which may be explained in part by disparities in sample size, population, and ethnicity, warrants further studies on large populations of diverse ethnic groups.

**Fig. 1:**
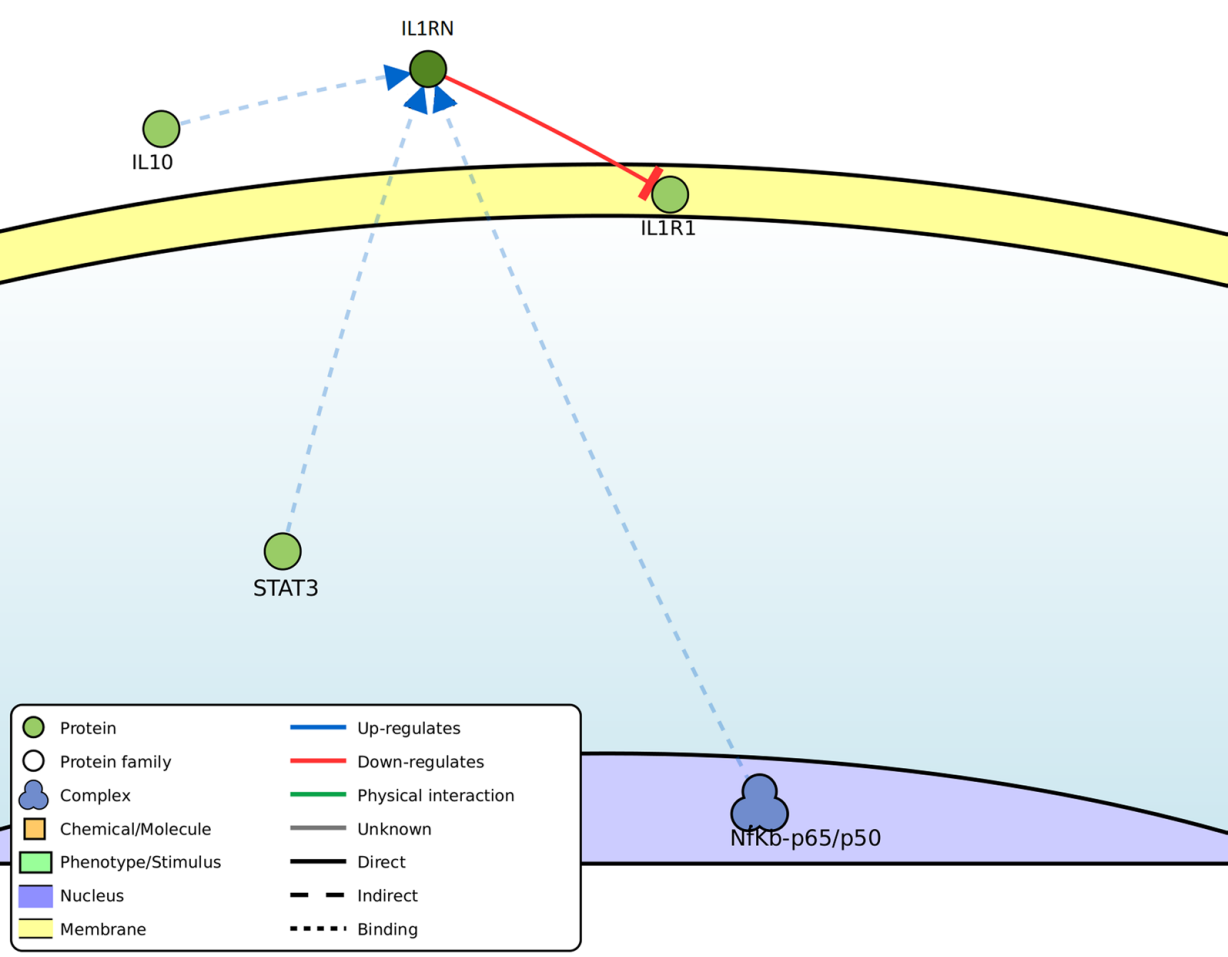
Regulatory network of IL -1RN as predicted by Signor (https://signor.uniroma2.it/); IL -1RN is positively upregulated by NFκb-p65/p50, STAT3, and IL-10

The IL-1RN protein is an important negative regulator of the inflammatory response. This gene, which belongs to the IL-1 cytokine family, hinders the function of IL-1 (IL-1α and IL-1β) by interacting with the receptor IL-1R1 and keeping the coreceptor IL-1RAP (IL-1 receptor accessory protein) from associating with it for signal transduction (Fig. 1). As predicted by Signor (https://signor.uniroma2.it/), the gene is positively upregulated by NFκb- p65/p50, STAT3, and IL -10 (Fig. 1). In fact, IL-10 activates STAT3, triggering it to make the IL-1RN promoter accessible to nuclear NF-κB [31]. This highlights the value of further investigation of this pathway for future studies on KC.

IL-1β is located in the IL-1 gene cluster and contains putative functional polymorphisms associated with various human diseases [32]. The two IL -1β SNPs studied here were rs16944, C > T, and rs1143634, C > T. Our current findings on the role of rs16944 in KC is controversial; a study on an Egyptian population found the C allele to be associated with greater risk of KC [33], while in another study on the Chinese Han population, the carriers of the T allele had greater risk for KC [34]. Additionally, Palmar et al. reported no significant association between rs16944 genotypes and no susceptibility to KC was detected [30]. Although we did not detect a significant correlation between the genotype and allele frequencies of the IL-1 rs16944 variant and KC risk, it was associated with KC risk in the form of the CCL (haplotype h2) and TCS (haplotype h7) haplotypes. This observation underscores the importance of considering the role of each genetic variant, not as individual players, but as nodes in an intertwined pathway.

The variant IL-1β rs16944, C > T, is located at position −511 within the promoter region. Regulatory sequence variations can strongly affect the expression levels of their corresponding gene. Supporting evidence is available in the case of rs16944, where the T allele of IL-1β at position −511 has been reported to result in modestly increased transcriptional activity [29, 35]. Furthermore, homozygosity for the T allele at −511 and the C allele at −31 is correlated with increased IL-1β secretor phenotype [33]. The expression of IL-1β has been elevated in KC compared to normal cornea [36]. The second IL-1β polymorphism studied here is rs1143634, C > T. This SNP is located at the exon 5 position + 3954 and is considered a synonymous variant (NP_000567.1:p.Phe105 exon 5 =). This variant was previously associated with the risk of disorders like gastric cancer, endometriosis, and non-Sjogren dry eye [37–39]. There are a few reports on rs1143634, C > T, and susceptibility to KC. According to our results, the existence of the rs1143634 T allele could increase the risk of KC by 1.53 fold. In addition, the genotype TC could significantly increase the risk of KC in dominant and codominant modes of inheritance. However, in the Korean population, rs1143634 has not been associated with the risk of KC. The contradictory results again emphasize the significance of additional investigations in larger populations across various ethnic backgrounds.

Clinical applications including the diagnosis, treatment, and management of KC hold great promise based on the genetic results of this study. The discovery of certain IL-1 gene cluster variations linked to an increased risk of developing KC may result in the developing of genetic screening instruments that assist in identifying people who are more susceptible to the disease, which allows for earlier and more focused therapies. Furthermore, by knowing the genetic foundation of KC, we can advance customized medicine strategies that modify treatment according to a patient's genetic profile. For example, people with particular genetic markers could benefit from targeted therapy approaches meant to modify the inflammatory processes linked to KC.

It is important to recognize the limitations of this study. Firstly, corneal epithelial mapping is not included in the Pentacam equipment used in this study, despite the fact that epithelial thickness mapping is essential for KC diagnosis, especially in the early stages. Due to this restriction, occasional false-positive KC diagnoses may result, particularly in situations with anterior basement membrane dystrophy. Other than that, despite its large sample size, the study may not adequately reflect the genetic diversity of the Iranian population as a whole. Additional studies using greater and more varied cohorts are required to corroborate these results and investigate the mechanisms at work. Lastly, bias may have been introduced since environmental variables that are known to affect the development of KC, including wearing contact lenses and scratching one's eyes, were self-reported rather than objectively evaluated.

## Conclusion

KC is a multifactorial disease involving both genetic and environmental components. Understanding the underlying genetic factors related to the predisposition or susceptibility can play an important role in the pathogenesis, prognostic evaluation and management. The current study demonstrates a strong association between IL-1β and IL-1RN variants and exposure to KC in Iran.

## Supplementary Information

Additional file 1.

## Data Availability

All data generated or analyzed during this study are included in this published article and its supplementary information files.

## Abbreviations

KC: Keratoconus
IL -1: Interleukin-1
MMP: Matrix metallopeptidase
LD: Linkage disequilibrium
PCR: Polymerase chain reaction
RFLP: Restriction fragment length polymorphism
SNP: Single nucleotide polymorphism
OR: Odds ratio
CI: Confidence interval
SD: Standard deviation
HWE: Hardy–Weinberg equilibrium
LOD: Logarithm of the odds
