# Supplementary material for: Inflammatory and genomic interactions within keratoconus susceptible patients: a nationwide registered case–control study
Source: Eye Vis (Lond). 2024 Oct 2;11:40. doi: 10.1186/s40662-024-00407-z (PMC11446043; doi:10.1186/s40662-024-00407-z)
Supplement: Supplementary file 1 — Additional file 1. [file 40662_2024_407_MOESM1_ESM.docx]

**Supplementary Figure 1**


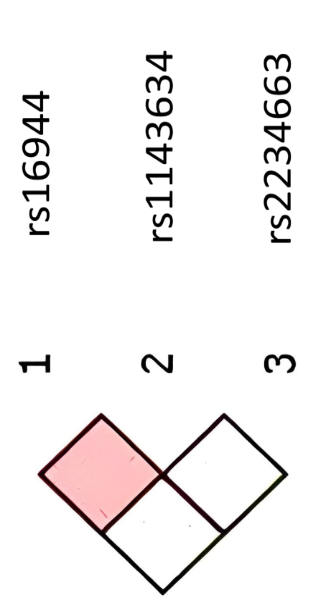


**Supplementary Table 1.** Pentacam criteria for risk of keratectasia.

| **Pentacam criteria** | **Normal** | **Suspect** | **Abnormal** |
| --- | --- | --- | --- |
| Kmax (D) | <47.2 | 47.2–49 | >49 |
| Against the rule astigmatism (D) | <1 | 1–2 | >2 |
| Corneal astigmatism (D) | <6 | 6–7 | >7 |
| Thinnest point (μm) | >500 | 470–500 | <470 |
| Difference between pachy apex and thinnest location (μm) | <10 | 10–20 | >20 |
| Difference central thickness between two eyes (μm) | <10 | 10–30 | >30 |
| Displacement of the thinnest point from the center (mm) | <0.5 | 0.5–1 | >1 |
| Skewed steepest radial axis (SRAX) (degrees) | <10 | 10–20 | >21 |
| IS value (inferior-superior difference at the 3 mm) (D) | <1.4 | 1.4–1.9 | >1.9 |
| IS value (inferior-superior difference at the 5 mm) (D) | <1.4 | 1.4–2.5 | >2.5 |
| Anterior elevation (μm) | <10 | 10–12 | >12 |
| Posterior elevation (μm) | <15 | 15–17 | >17 |

**Supplementary Table 2.** Clinically suggested cut-off values for keratoconus indices in screening clinical and subclinical cases.

| Parameter | Clinical keratoconus | Subclinical keratoconus |
| --- | --- | --- |
| Tomographic | | |
| CKI | ** | ** |
| KI | 1.07 | ** |
| IHA | 10.4 | ** |
| IHD | 0.017 | ** |
| TKC | 1.00 | 2.00 |
| ISV | 36.6 | ** |
| IVA | 0.28 | 0.15 |
| Rmin | 7.04 | ** |
| PE | 20.5 | 10.5 |
| IS value | 1.1 | 1.9 |
| KISA | 60% | 100% |
| Pachymetric | | |
| ART-Min | 606 | ** |
| ART-Max | 356 | 368 |
| ART-Avg | 444 | 490 |
| BAD_D | 2.02 | 1.31 |
| CCT | 515 | 518 |
| PPI-Min | 0.87 | 0.80 |
| PPI-Max | 1.53 | 1.40 |
| PPI-Avg | 1.18 | 1.08 |
| TCT | 506 | 502 |

CKI = central keratoconus index; KI = keratoconus index; IHA = index of height asymmetry; IHD = index of height decentration; TKC = topographic keratoconus classification; ISV = index surface variance; IVA = index of vertical asymmetry; Rmin = minimum radius of curvature; PE = prediction error; IS value = the inferior-superior value; ART = Ambrosio's relational thickness indices; BAD_D = Belin/Ambrósio deviation; CCT = central corneal thickness; PPI = pachymetric progression indices; TCT = thinnest corneal thickness; KISA: The KISA index was derived from the following 4 indices: central keratometry (K); I–S; the astigmatism index (AST), which quantifies the degree of the regular corneal astigmatism (simulated K1 − simulated K2); and the SRAX index, an expression of irregular astigmatism occurring in keratoconus.

**Although studies have addressed this topic, no specific figure has been established due to differing opinions and varying cut-off points

**Supplementary Table 3.** Specific forward and reverse primers, polymerase chain reaction (PCR) products, and digested products for genotyping of rs2234663, rs1143634, and rs16944 polymorphisms by PCR-restriction-fragment length polymorphism (PCR-RFLP).

| **SNPs** | **Primer sequences (5’🡪3’)** | **PCR product size (bp)** | **Digested fragments (bp)** |
| --- | --- | --- | --- |
| rs2234663 | Forward: CTCAGCAACACTCCTAT | 1 repeat: 154  2 repeats: 240  3 repeats: 326  4 repeats: 412  5 repeats: 498  6 repeats: 584 | - |
|  | Reverse: TCCTGGTCTGCAGGTAA |  |  |
| rs1143634 | Forward: GAGCGTGCAGTTCAGTGATC | 240 | TT: 240  TC: 240, 178, 62  CC: 178, 62 |
|  | Reverse: TGTTCTTAGCCACCCCACTC |  |  |
| rs16944 | Forward: CGTTGTGCAGTTGATGTCCA | 399 | CC: 254,145  TC: 399, 245, 145  TT: 399 |
|  | Reverse: CGTTGTGCAGTTGATGTCCA |  |  |

SNPs = single nucleotide polymorphisms
